# Light-induced chromophore and protein responses and mechanical signal transduction of BLUF proteins

**DOI:** 10.1007/s12551-017-0355-6

**Published:** 2017-12-12

**Authors:** Tomotsumi Fujisawa, Shinji Masuda

**Affiliations:** 1 Department of Chemistry, Graduate School of Science and Engineering, Saga University, Saga, 840-8502 Japan; 2 Center for Biological Resources & Informatics, Tokyo Institute of Technology, Yokohama, 226-8501 Japan

**Keywords:** Blue light–using flavin protein, Flavin, Photoreceptor, Phototaxis, Signal transduction, Synechocystis

## Abstract

Photoreceptor proteins have been used to study how protein conformational changes are induced by alterations in their environments and how their signals are transmitted to downstream factors to dictate physiological responses. These proteins are attractive models because their signal transduction aspects and structural changes can be precisely regulated in vivo and in vitro based on light intensity. Among the known photoreceptors, members of the blue light–using flavin (BLUF) protein family have been well characterized with regard to how they control various light-dependent physiological responses in several microorganisms. Herein, we summarize our current understanding of their photoactivation and signal-transduction mechanisms. For signal transduction, we review recent studies concerning how the BLUF protein, PixD, transmits a light-induced signal to its downstream factor, PixE, to modulate phototaxis of the cyanobacterium *Synechocystis* sp. PCC6803.

## Introduction

Most organisms have evolved light-sensory systems that optimize cellular and physiological responses, including photosynthesis and development. Various types of photosensory proteins, also denoted photoreceptors, have been identified in different organisms (Moglich et al. 2010). These photoreceptors bind various types of chromophores that absorb light energy of different wavelengths and thereby convert the absorbed energy into conformational changes by various mechanisms. Blue light–using flavin (BLUF) proteins, which are found in many bacteria and certain algae, are photoreceptors that contain a flavin chromophore that absorbs blue light (Gomelsky and Klug 2002; Masuda 2013; Conrad et al. 2014; Mathes and Gotze 2015). The chromophore-binding domain (~15 kDa), denoted as the BLUF domain, is present in single- and multi-domain proteins, and it transmits the light-induced signal to downstream protein modules via intermolecular or intramolecular interactions. Unlike many other types of photoreceptors, e.g., those containing rhodopsin and phytochrome in which the chromophore undergoes a large conformational change when irradiated (e.g., *trans-cis* isomerization) to activate the light-signaling state (Moglich et al. 2010), the photoactivation mechanisms of BLUF proteins are not accompanied by major structural changes in the chromophore. Consequently, the mechanism by which the flavin transmits the light signal to the apoprotein and then downstream has generated considerable interest. Herein, we summarize our current understanding of the photoactivation mechanism of the BLUF domain**,** and the intra- and intermolecular light-signal transduction of the BLUF protein PixD that controls the phototaxis response of the cyanobacterium *Synechocystis* sp. PCC6803.

## Photoactivation of BLUF domains: The molecular mechanism

**Fig. 1:**
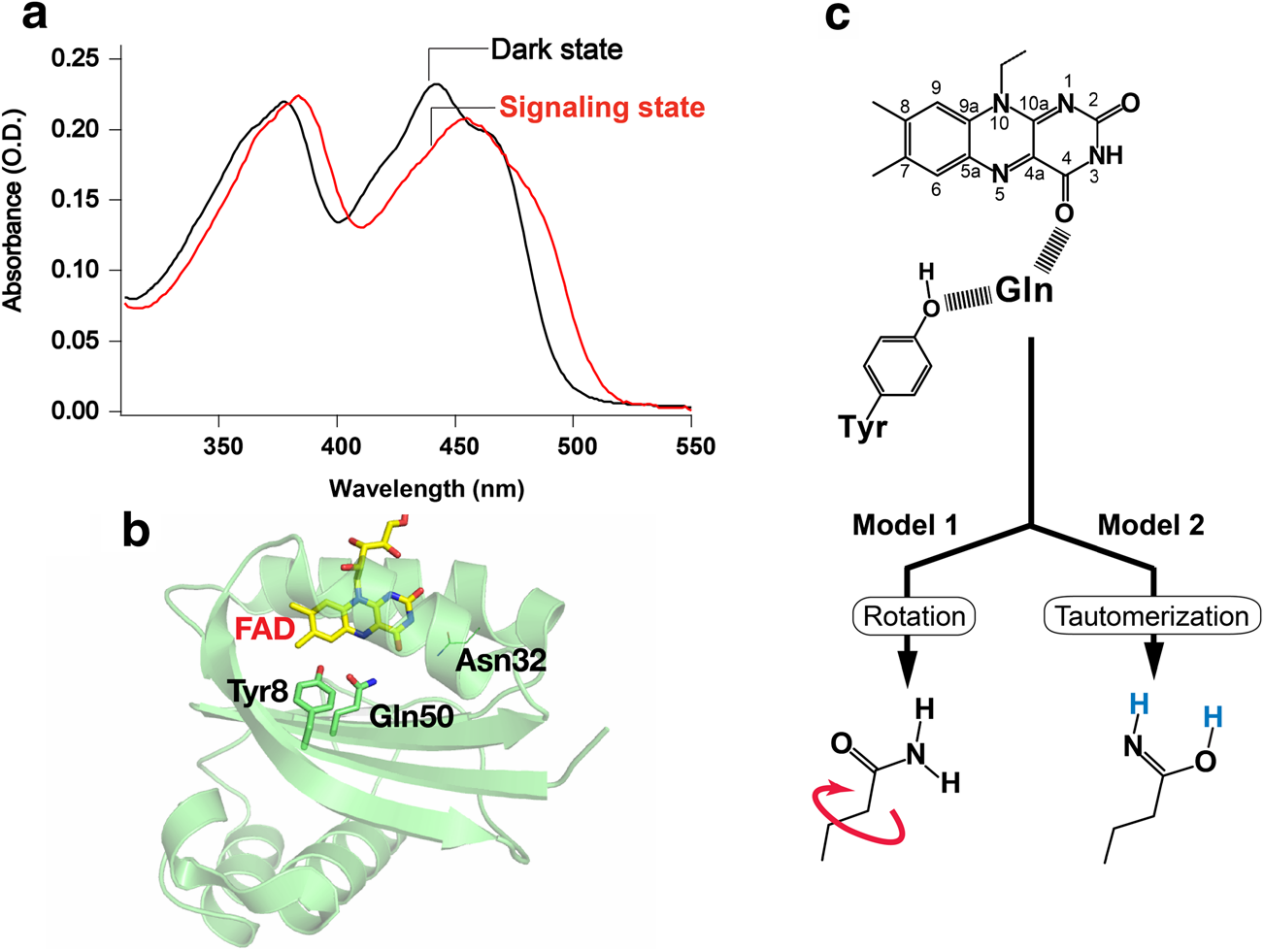
**a** Absorption spectra for PixD in its dark and signaling states. **b** Ribbon diagram of the PixD crystal structure (PDB entry: 2HFO) with the FAD chromophore and nearby conserved residues illustrated as stick models. **c** Chemical or mechanical changes proposed for the Gln side chain accompanying the hydrogen-bond rearrangement in the FAD-Gln-Tyr triad. *Model 1*: rotation of Gln; *Model 2*: keto-enol tautomerization of Gln

Figure 1a illustrates the absorption spectra of PixD in its dark and signaling states (i.e., before and after the light illumination). These absorption spectra inform that both states contain the same flavin chromophore, FAD (flavin adenine dinucleotide), but the ~10 nm redshift found for the signaling state indicates that a new interaction(s) between the chromophore and the apoprotein leads to the photoactivation. The mechanism of this distinct signaling-state formation has attracted considerable interest from experimental and theoretical studies and, at the same time, it has also provoked significant controversy. Currently, it is commonly recognized that the signaling state of BLUF proteins arises from the structural change of the hydrogen bond network between FAD, Gln, and Tyr in the active site (Masuda 2013; Moglich et al. 2010; Zoltowski and Gardner 2011), where the key player is the Gln side chain that undergoes chemical and/or mechanical changes between the FAD and Tyr.

### Structure of the active site

In the active sites of BLUF proteins, the FAD chromophore is non-covalently bound to the protein, with hydrophobic contacts as well as hydrogen bond interactions formed with several amino acid residues. The strictly conserved residues that can make the hydrogen bonds with the chromophore include Gln and Asn (Fig. 1b, Gln50 and Asn32 in PixD), which interact with the C_4_ = O carbonyl group of FAD. Specifically, the Gln also makes a hydrogen bond with the phenolic hydroxyl of a conserved Tyr (Tyr8 in PixD) so as to form an FAD–Gln–Tyr hydrogen-bond network. The early spectroscopic analyses of BLUF proteins observed a ~20 cm^−1^ downshift of the C_4_ = O stretch of the FAD chromophore in the signaling state, and the downshift indicated that the stronger hydrogen bonding, which is created around the C_4_ = O carbonyl of FAD, give rise to the signaling state (Laan et al. 2003; Masuda et al. 2004; Unno et al. 2005). Before the crystal structure had been solved, the direct interaction between the conserved Tyr and FAD in the signaling state was temporarily suggested because the mutation of the Tyr abolished the light-induced ~10 nm redshift (Kraft et al. 2003; Laan et al. 2003). Thereafter, the X-ray crystallography disclosed that the amino acid residue directly interacting with C_4_ = O carbonyl of the chromophore was not the Tyr but the Gln and Asn (Anderson et al. 2005; Jung et al. 2005; Kita et al. 2005; Yuan et al. 2006). Importantly, a mutagenesis study revealed that replacement of the Asn did not abolish the photoactivity of the BLUF protein, whereas mutating the Gln did abolish the photoactivation (Kita et al. 2005). The rearrangement of the hydrogen-bond network involving only the Tyr, Gln, and FAD was, therefore, recognized as essential for formation of a BLUF protein signaling state.

Although the Gln plays the critical role in the hydrogen bond structural change of FAD-Gln-Tyr, it has been nearly impossible to directly visualize the hydrogen bond network of FAD-Gln-Tyr in the dark and signaling states of BLUF proteins. For instance, the identification of the hydrogen-atom positions is not feasible by X-ray crystallography with a normal spatial resolution (~2 Å). Neither is the determination of the Gln orientation because the electron densities of the nitrogen atom of NH_2_ amino and oxygen atom of C = O carbonyl of the Gln side chain are similar. Therefore, this ambiguity about the hydrogen bond structure spurred the intense debate centered on the two models that describe the possible rearrangement of the FAD–Gln–Tyr hydrogen-bond network, as shown in Fig. 1c. The first model (model 1) is the rotation of the Gln which was proposed by X-ray crystallography (Anderson et al. 2005), postulating that the hydrogen-bond rearrangement occurs as a consequence of a rotation of the Gln. On the other hand, the theoretical studies predicted the second model (model 2), for which the rearrangement of the hydrogen-bond network is induced by a keto-enol tautomerization of the Gln side-chain carbonyl with the enol tautomeric state (Domratcheva et al. 2008; Sadeghian et al. 2008). The work of Sadeghian and colleagues supported the keto-enol tautomerization model that does not involve rotation of the Gln (Sadeghian et al. 2008), whereas Domratcheva and coworkers predicted that the tautomerization would be accompanied by rotation of the Gln (Domratcheva et al. 2008; Khrenova et al. 2013; Udvarhelyi and Domratcheva 2013). These models based on the keto-enol tautomerization, however, were not readily accepted because the strong experimental support was not obtained; the structural data by Raman and NMR spectroscopies were explained without the consideration of the Gln tautormerization (Grinstead et al. 2006a, b; Unno et al. 2006). Only recently a Fourier-transform infrared (FTIR) difference spectral study by Domratcheva and co-workers provided experimental evidence for the Gln tautomerization. That study used a BLUF protein labeled with ^15^N–labeled Gln to extract the structural change of the Gln from the light-induced FTIR difference spectrum. Then, they showed that the structural change of the Gln between the dark and signaling states was not explained well by the rotation of the Gln keto form, rather it was reproduced by the keto-enol tautomerization with the aid of quantum chemical calculations (Domratcheva et al. 2016). Now, the keto-enol tautomerization of the Gln has become the consensus model.

In both the dark and signaling states of BLUF proteins, it is not very clear whether the C_4_ = O carbonyl of FAD interacts with NH_2_ amino (or N-H imine) or C = O keto (or O-H enol) group of Gln. Actually, there was a contradiction about the orientation of the Gln side chain in the crystal structures of the dark state (Anderson et al. 2005; Jung et al. 2006). Related to this issue, theoretical calculations indicated that the energetically preferred dark state involves a hydrogen bond between the C_4_ = O carbonyl of FAD and the Gln amino group but the rotation of Gln side chain may also occur because of the low activation energy (Udvarhelyi and Domratcheva 2013). For the hydrogen bond structure in the signaling state, the FTIR difference spectroscopy in high frequency region (2000–3500 cm^−1^) made an important observation (Iwata et al. 2011). The FTIR measurement revealed the unusual downshift of the OH stretch of the strictly-conserved Tyr in the active site of the signaling state, indicating that the Gln also forms the very strong hydrogen bond with the Tyr on the opposite side of FAD (Iwata et al. 2011). The theoretical calculation suggested that this large downshift of OH stretching frequency is reproduced when the OH group of Tyr is hydrogen bonded to N-H imine of the Gln concurrently with the enol OH of the Gln hydrogen bonding with the C_4_ = O carbonyl of FAD (Domratcheva et al. 2016). Therefore, the theoretical calculation suggested that the Gln rotates to form the hydrogen bond between its enol OH and FAD in the signaling state (Domratcheva et al. 2016; Udvarhelyi and Domratcheva 2013); however, experimental evidence for the Gln rotation has yet to be obtained.

### Photochemical process

Flavin is a fluorescent molecule that is not photoreactive by itself. In protein, however, the fluorescence quantum yield of the flavin cofactor often becomes much lower because the photoinduced reaction with the surrounding amino acid residue(s) quenches the excited state (Kao et al. 2008; Liu et al. 2013; Mataga et al. 2000). The photoreaction of flavin is, in many cases, observed as the light-induced redox reactions with the aromatic amino acids involving the transfers of proton and electron in picosecond timescale (Kao et al. 2008; Mataga et al. 2000; Tanaka et al. 2007). In BLUF proteins as well, the low fluorescence quantum yields of the FAD chromophore (Tyagi et al. 2008; Zirak et al. 2007; Zirak et al. 2005, 2006) is indicative of a photochemical reaction, which lies behind the hydrogen-bond structural change in the active site (Conrad et al. 2014; Kennis and Mathes 2013; Mathes and Gotze 2015).

**Fig. 2:**
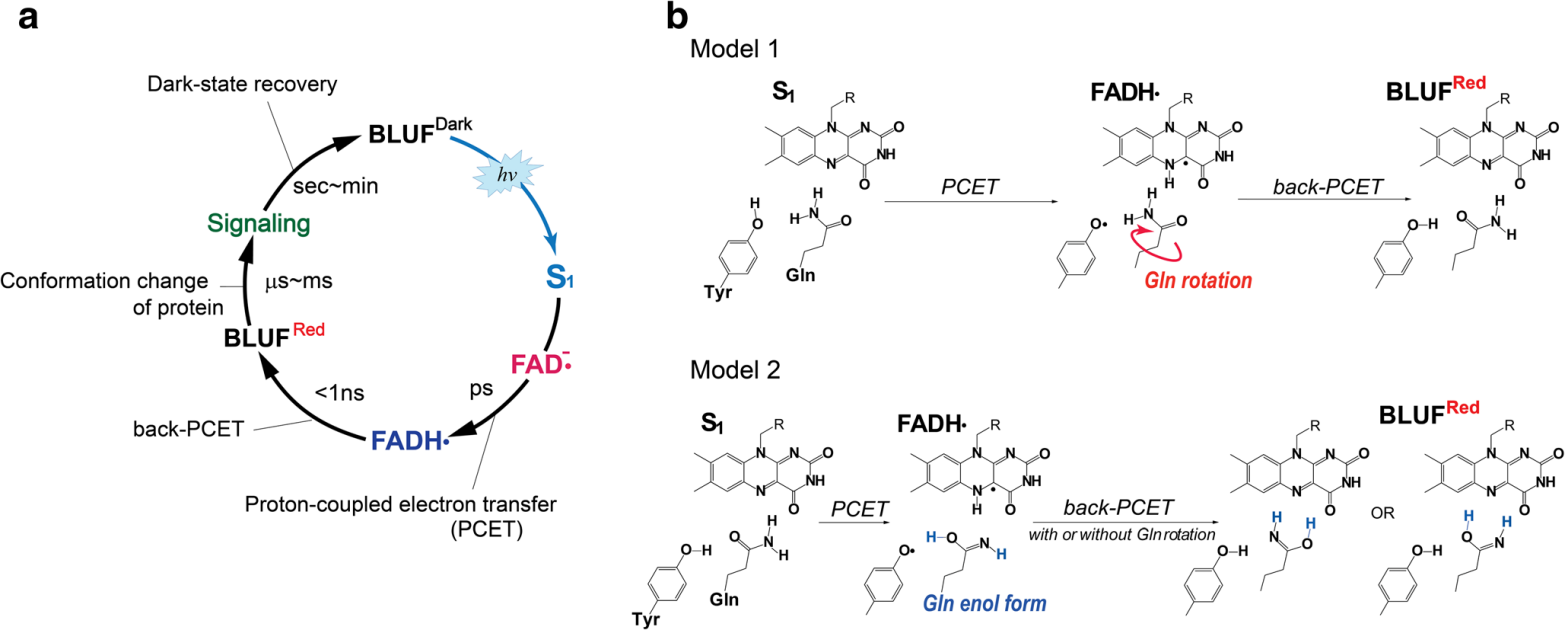
**a** Representative BLUF protein photocycle. **b** Proposed hydrogen-bond rearrangement process. *Models 1 and 2* involve a rotation of the keto form of Gln and keto-enol tautomerization of Gln, respectively

BLUF proteins exhibit a light-induced cyclic reaction, i.e., a photocycle, as the photoexciation of the protein induces the signaling state, which then thermally returns to the original dark state. The initial photoreaction of PixD occurs on the picosecond time scale, as shown by the time-resolved absorption study of Kennis and coworkers (Bonetti et al. 2009; Gauden et al. 2006). After the photoexcitation of PixD, the FAD semiquinone radical (FADH•) was produced from the first singlet excited state (S_1_ state) in several tens of picoseconds; then PixD^Red^, which shows the redshifted absorption band, was generated within 1 ns after the decay of the FADH•. This photoreaction process of PixD (Fig. 2a) first demonstrated the involvement of proton-coupled electron transfer in the hydrogen-bond rearrangement of FAD-Gln-Tyr triad. The donor of an electron and proton during FADH• formation is most likely the conserved Tyr, with the proton transfer taking place via the FAD–Gln–Tyr hydrogen-bond network. In a time-resolved absorption study of the BLUF protein, AppA from *Rhodobacter sphaeroides*, an intermediate reaction was not observed before the AppA^Red^ was produced (Dragnea et al. 2005; Gauden et al. 2005). The appearance of FADH• in the PixD photocycle was attributed to its relatively long lifetime. On the basis of the photoreaction of PixD, the Gln rotation was proposed to occur in the presence of the FADH•-Tyr• radical pair, as illustrated in model 1 (Fig. 2b) (Bonetti et al. 2008). Meanwhile, theoretical studies predicted the different model based on keto-enol tautomerization of the Gln (Domratcheva et al. 2008; Sadeghian et al. 2008). According to this model 2 (Fig. 2b), the enol form of the Gln is produced during formation of FADH•, and the Gln may rotate after the production of FADH•. In either experimental or theoretical studies (Bonetti et al. 2009; Gauden et al. 2006; Mathes et al. 2012a), FADH• has been commonly considered as the important reaction intermediate for the change of the FAD-Gln-Tyr hydrogen bond network. Currently, model 2, involving keto-enol tautomerization, has obtained both experimental and theoretical support as mentioned previously, but the dynamical aspect still remains to be proven.

To date, the dynamic process for the hydrogen-bond rearrangement of the FAD–Gln–Tyr has been investigated by ultrafast time-resolved spectroscopic techniques. Femtosecond IR spectroscopy has proved to be a powerful method to observe ultrafast dynamics of the chromophore and the protein structures of BLUF proteins (Brust et al. 2013, 2014; Lukacs et al. 2011, 2014; Stelling et al. 2007). In the time-resolved femtosecond IR spectra of BLUF proteins, the excited-state vibrational bands as well as the ground-state bleach of the chromophore appear immediately after photoexcitation (Brust et al. 2013; Lukacs et al. 2014). Subsequently, a small vibrational band attributed to FADH• was observed when PixD was photoexcited (Lukacs et al. 2014). These observed vibrational bands were assigned in detail by isotope-labeling of the chromophore (Brust et al. 2013; Haigney et al. 2011, 2012; Lukacs et al. 2014). However, the clear vibrational modes of the Gln or the marker bands for the FAD–Gln–Tyr hydrogen bonding have not been identified in the time-resolved spectrum (Lukacs et al. 2011; Stelling et al. 2007).

Different attempts to study the hydrogen-bond dynamics of the FAD–Gln–Tyr have been made by femtosecond time-resolved absorption studies (Fujisawa et al. 2014; Mathes et al. 2012b; Toh et al. 2008). Although time-resolved electronic spectroscopy is intrinsically insensitive to the structural dynamics of the FAD chromophore, the reaction process is affected by the protein environment around the FAD chromophore and can, therefore, potentially provide the dynamical structural information concerning the active site. For BLUF proteins, the kinetics of FADH• was used to probe the hydrogen bonds of the FAD–Gln–Tyr because the formation/decay of FADH• involves a proton transfer via the FAD–Gln–Tyr hydrogen-bond network. Currently, two BLUF proteins, PixD and PapB (from *Rhodopseudomonas palustris*), were found to sufficiently produce FADH• as the reaction intermediate in their photoreactions (Fujisawa et al. 2014; Mathes et al. 2012b). The time-resolved absorption study of the Trp91→Phe mutant of PixD observed the production of FADH• after exciting either the dark or signaling state. Kinetic analysis revealed that FADH• was formed in the heterogeneous environment of the active site, and the faster formation time of FADH• generated from the signaling state was interpreted as a stronger FAD–Gln–Tyr hydrogen-bond network that facilitates proton transfer within the FAD–Gln–Tyr (Mathes et al. 2012b). Conversely, PapB showed the same FADH• kinetics produced from the dark and signaling states within the experimental uncertainty. This result suggested that the hydrogen-bond structure of the FAD–Gln–Tyr in the dark and signaling states were already indistinguishable before the FADH• formation, which in turn suggests that a structural change in the FAD-Gln-Tyr triad occurs before formation of FADH• (Fujisawa et al. 2014). However, despite the aforementioned ultrafast electronic and vibrational spectroscopic studies, only indirect information was obtained for the hydrogen bond rearrangement process in BLUF proteins. The experimental studies of the ultrafast dynamics of BLUF proteins have been facing the challenge to verify the process of FAD–Gln–Tyr rearrangement, for achieving the mutual agreement between the experimental observation and the theoretical predictions.

### Protein dynamics

During photoactivation from the dark to signaling state of a BLUF protein, the hydrogen-bond structural change in the active site propagates through the protein and alters its conformation to transmit the light-induced allosteric signal. The conformations of its C-terminal helices are particularly relevant to signal transduction in PixD. Then, the intramolecular conformational changes can even control the interprotein association or dissociation that regulates the activities of the protein. The protein dynamics that follow the ultrafast photoreaction have been studied by time-resolved spectroscopy in the microsecond-to-millisecond region. Time-resolved IR spectroscopy was used to study the photo-induced dynamics of the secondary structure in the BLUF domain, which revealed that the structural change in the β-sheet occurred within 20 μs (Brust et al. 2013). Additional spectral changes related to protein dynamics in the millisecond range were mostly absent, although Terazima and coworkers identified a temporal change in the higher-order structure with the use of a transient-grating technique (Hazra et al. 2006; Nakasone et al. 2007, 2010; Tanaka et al. 2009, 2011). This spectroscopic method detects diffusion of photoactive proteins in real time, a process that is sensitive to their association/dissociation dynamics (Terazima 2011). For the PixD, the oligomeric dark state was revealed to dissociate into dimeric light state with the time constant of 350 ms after photoexcitation (Tanaka et al. 2011).

**Fig. 3:**
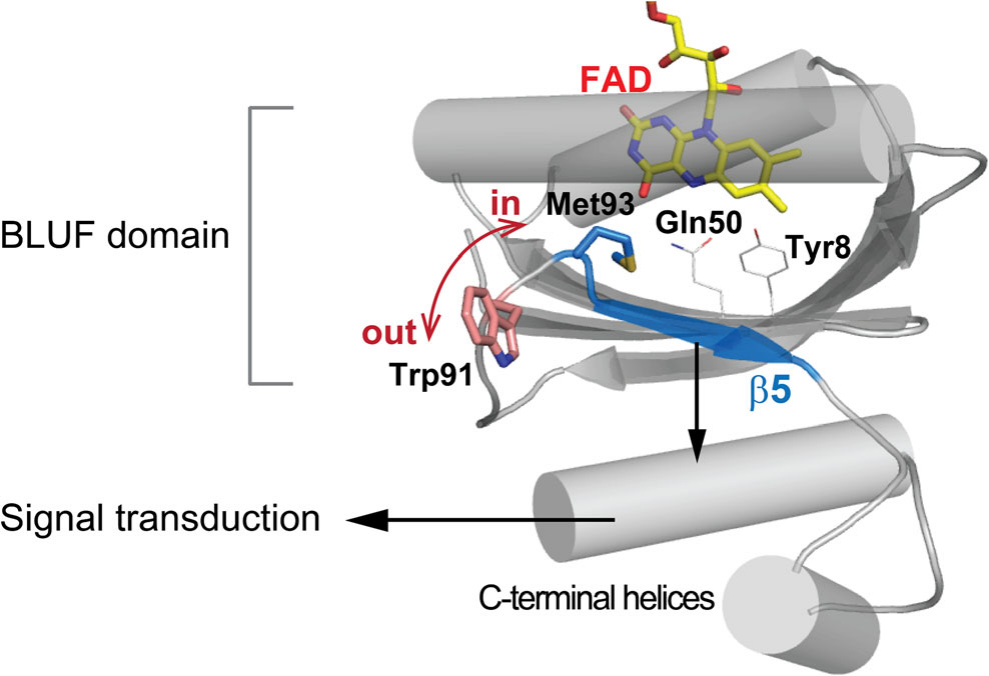
Structure of PixD (PDB entry: 2HFO) with the residues involved in the dark-to-signaling state conformational changes shown as stick models

The methionine residue, in the fifth β strand, is highly conserved and has an important role in the conformational change that occurs upon photoactivation (Masuda et al. 2008). As shown in Fig. 3, the Met93 in PixD is positioned near the Gln50 so that the two residues can form a hydrogen bond. When the Met93 was replaced with an alanine (i.e., M93A mutant), the light-induced conformational change in the amide-II region in the FTIR difference spectrum was absent even though the redshift of the chromophore in the absorption spectrum, which is a signature of the hydrogen-bond rearrangement in the FAD–Gln–Tyr triad, was present (Masuda et al. 2008). Therefore, Met93 appears to transfer the structural change at the active site into the conformational change involving the C-terminal helices (Fig. 3). In addition, a *Synechocystis* strain expressing the M93A mutant exhibited negative phototaxis (i.e., movement away from the light source) as opposed to the positive phototaxis of the wild-type strain. This finding indicated that the M93A mutant is functionally locked into the signaling state, which implies that loss of the hydrogen bond between the Gln and the Met may cause a conformation change in BLUF proteins (Masuda et al. 2008).

The semiconserved tryptophan residue near the methionine is another amino acid residue whose involvement in the signaling state formation has been studied (Dragnea et al. 2009; Jung et al. 2006; Masuda et al. 2005b, 2007; Mehlhorn et al. 2015; Unno et al. 2010). The relevance of the Trp104 to the induction of the signaling state was first demonstrated for AppA (Masuda et al. 2005b); the light-induced FTIR difference spectroscopy of AppA showed that the protein structural change reflected in the amide I region vanished when the Trp is mutated by Ala. (Masuda et al. 2005b). A subsequent in-vivo and in-vitro site-directed mutagenesis study revealed that Trp104 is crucial for the biochemical function of AppA (Masuda et al. 2007). X-ray crystallography (Jung et al. 2006) suggested that Trp104 in the dark state adopted an orientation denoted Trp^in^ whose indole side chain pointed toward the FAD-binding site in the protein interior, whereas it adopted Trp^out^ conformation in the signaling state as the aromatic side chain was oriented toward protein surface (cf. Fig. 3). Later, this model was challenged by the results of fluorescence-quenching and UV Raman spectral studies (Dragnea et al. 2009; Toh et al. 2008; Unno et al. 2010) which indicated that the movement of the tryptophan was not as drastic and that it was buried within the interior of protein in both dark and signaling state. In contrast, the corresponding tryptophan at 91 position (Trp91) in PixD was found to be less important for the light-induced conformational change when compared to Trp104 in AppA. Mutation of Trp91 to an alanine in PixD did not significantly change its light-induced FTIR difference spectrum, as well as the macroscopic phototactic response of the bacterium (Masuda et al. 2008; Mehlhorn et al. 2015). These results indicate that the light-induced conformational changes in BLUF proteins vary and that the conformational changes of PixD and other BLUF proteins likely respond to their particular signal-transduction requirements at the molecular level, even though their initial photoactivation mechanisms involve common chemical and structural changes at the FAD-binding site.

In comparison with photoactivation from the dark to signaling state, the reverse process, i.e., recovery of the dark state, has been much less examined. Recovery of the dark state occurs with a time constant of seconds to minutes, and the rate of its recovery is decreased in D_2_O — by a factor of four for PixD (Masuda et al. 2004) and ~two-fold for AppA (Laan et al. 2006; Masuda et al. 2005a) — indicating that the rate-limiting step involves a proton transfer. Recovery of the dark state is accelerated up to ~100-fold in the presence, at most, of 2.0 M imidazole (Laan et al. 2006). Further, recovery is affected by the mutation of the tryptophan residue near the active site: For PixD, the recovery of the dark state is accelerated in the Trp91→Ala on one hand, but it is slowed by a factor of 50 in the Trp91→Phe mutant on the other. (Mathes et al. 2012b; Mehlhorn et al. 2015). Recently, Agnieszka and co-workers showed that the recovery rate is linearly correlated with pKa values of the Tyr in the active site over a wide pKa range between 6 and 10 (Gil et al. 2016, 2017). They studied PixD and AppA in which the conserved Tyr21 was replaced by fluorotyrosines with the different pKa values, demonstrating clearly that the proton affinity of the Tyr was a crucial factor for the control of signaling-state stability or the rate of recovery to the dark state.

## PixD-dependent signal-relay mechanisms

Various combinations of BLUF domains and protein partners exist; e.g., photosynthesis-related gene expression in the purple bacterium *Rhodobacter sphaeroides*, biofilm formation in *Escherichia coli*, and the phototaxis response in the cyanobacterium *Synechocystis* sp. PCC6803 are controlled by AppA-PpsR, BluF-BluR, and PixD–PixE respectively. Given that a number of different proteins containing a BLUF domain and that their downstream partners vary, how the light-induced structural change(s) in their BLUF domains is transmitted downstream to control their physiological functions is an interesting issue. Next, we describe how PixD relays its light-dependent signal as an example of the intermolecular interactions between its BLUF domain and its downstream factor, PixE. Signal relays involving other BLUF domains and their downstream factors have been recently summarized (Lindner et al. 2015; Nudel and Hellingwerf 2015; Park and Tame 2017).

### Structure of the PixD oligomer

**Fig. 4:**
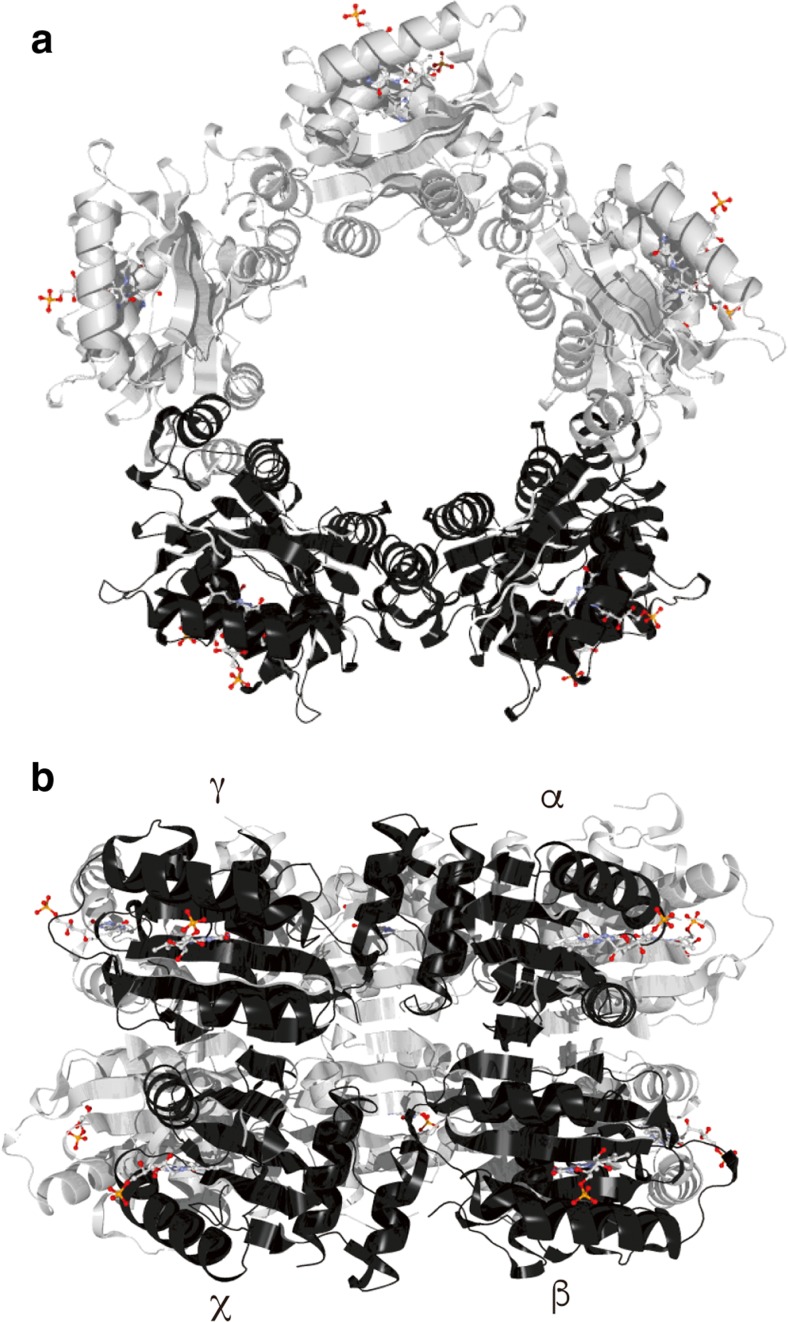
Top (**a**) and side (**b**) views of the PixD decamer crystal structure (PDB entry 2HFN). Four adjacent monomers, two each from one of the pentameric rings, are labeled α, β, γ, and χ. The flavin chromophores are indicated as ball-and-stick structures. Reproduced from (Ren et al. 2015) with a slight modification

Crystalline PixD forms a decamer in which two pentameric rings are stacked face-to-face (Yuan et al. 2006). Gel-filtration and blue-native PAGE studies have revealed that purified PixD equilibrates between a dimer and decamer in solution (Ren et al. 2013, 2015; Tanaka et al. 2011; Yuan and Bauer 2008), suggesting that the crystal structure of decameric PixD possibly reflects a decameric structure in vivo. Conversely, a solution structure for dimeric PixD has not been established. In decameric PixD, one PixD molecule (indicated as α in Fig. 4b) directly interacts with three other PixD molecules (indicated as β, γ, and χ in Fig. 4b), suggesting that PixD can potentially form three different dimeric states, i.e., e α-β, α-γ, and α-χ (Fig. 4b). Mino and co-workers used electron magnetic resonance spectroscopy to measure the distance and orientation of the FADH•-Tyr8• radical pair in dimeric PixD (Kondo et al. 2011a, b) and found that the PixD dimer in solution is most similar to that of the α–γ dimer in the crystal structure (Kondo et al. 2011a). The α–γ pair is formed between monomers within a pentameric ring, suggesting that decamer formation from a dimer would involve a large conformational change, e.g., dissociation of the molecules in the dimer and then association of five molecules to make a pentameric ring.

Ren and co-workers cross-linked purified PixD and examined the products by mass spectrometry (Ren et al. 2015). They found that two residues, Glu26 and Lys22, were cross-linked with a zero-length cross-linker to form a covalent dimer, suggesting that these two residues from different molecules are positioned closely in the PixD dimer. They also performed docking simulations to characterize possible intermolecular interactions in a dimeric PixD structure; their results indicated that Glu26 and Lys22 could be closely positioned and that the C-terminal regions of two PixD molecules can interact (Ren et al. 2015). These results underscore the importance of interactions between the C-terminus of two PixD molecules to form a dimer. When the C-terminal region of PixD is removed, PixD does not form a stable dimer, a finding that further supports the PixD dimeric structural model (Ren et al. 2015). The in-silico modeled PixD dimer is not similar to any dimeric combination found in the PixD crystal structure (Fig. 4), and its structure is also not consistent with the results of the electron magnetic resonance spectroscopy described above. Additional studies are necessary to establish the exact PixD dimeric structure. Nevertheless, formation of the PixD decamer from PixD dimers may involve large conformational re-arrangements of the PixD monomers.

Genetic analysis revealed that the response regulator-like protein PixE functionally interacts with PixD (Masuda et al. 2008; Okajima et al. 2005; Sugimoto et al. 2017). Yuan and co-workers reported that PixE promotes formation of the PixD decamer (Yuan and Bauer 2008). The C-terminal truncated version of PixD, which cannot form a stable dimer, also cannot form a stable PixD–PixE complex, although this truncated PixD can form an oligomer (perhaps a decamer) (Ren et al. 2015). These results suggest that PixE associates with the PixD dimer but not with the PixD decamer; indeed, the equilibrium between the PixD dimer and decamer determines the photosensitivity of the PixD-dependent light-signal transduction.

### Models for the PixD–PixE complex

**Fig. 5:**
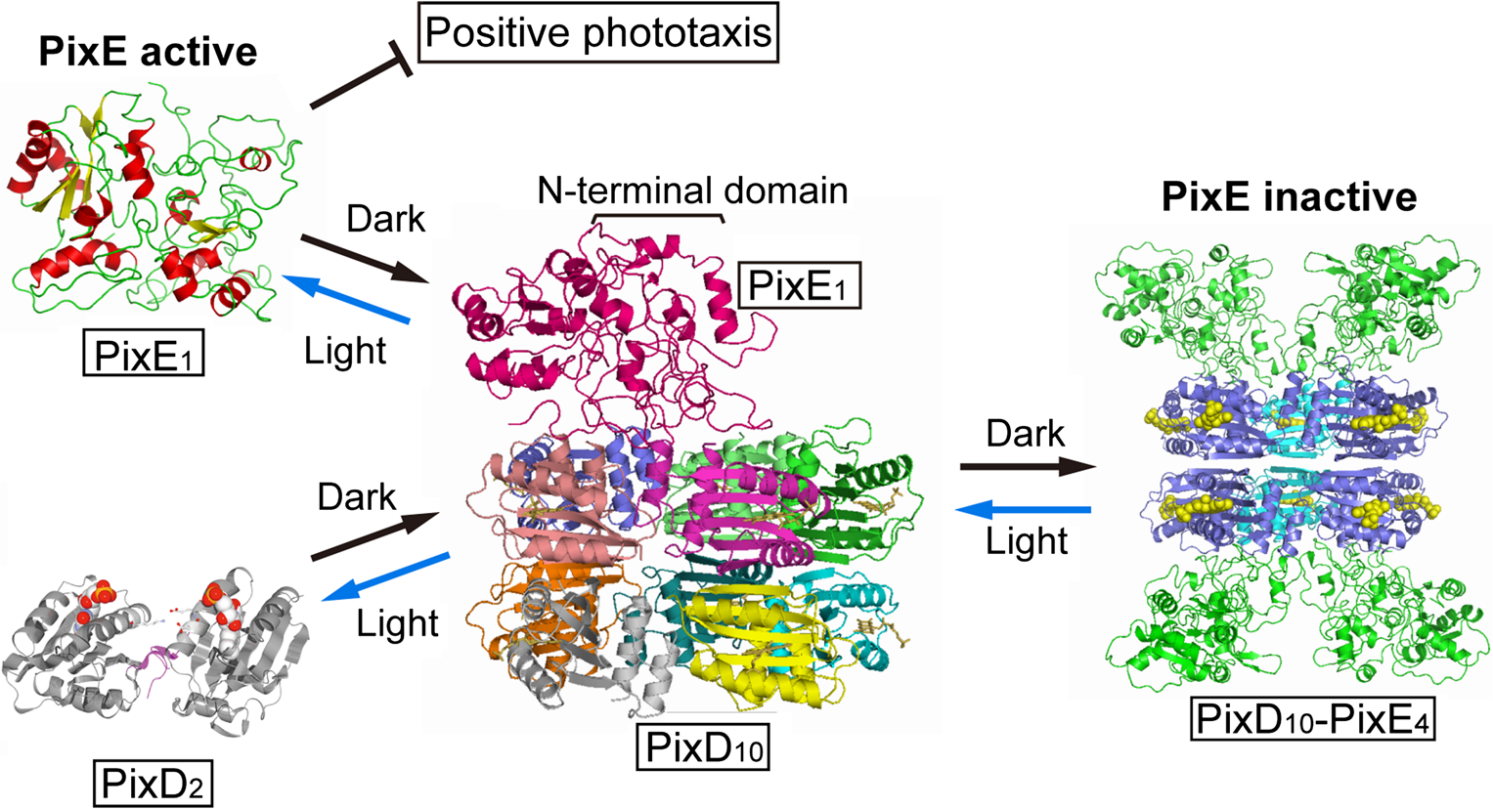
Schematic of how PixD and PixE control the phototaxis response of the cyanobacterium *Synechocystis* sp. PCC6803 In the dark, PixD forms a decamer and interacts with PixE to form a PixD_10_–PixE_4_ complex. Upon irradiation, PixD dissociates to form homodimers and PixE is released from the complex. The released monomeric PixE inhibits positive phototaxis. Reproduced from (Ren et al. 2015) with a slight modification

Two models for the PixD–PixE complex have been proposed; one involves a PixD decamer that interacts with five PixE monomers (PixD_10_–PixE_5_) (Tanaka et al. 2012; Yuan et al. 2006), and the other is a PixD decamer that interacts with four PixE monomers (PixD_10_–PixE_4_) (Fig. 5, (Ren et al. 2013). A size-exclusion chromatography study revealed that the PixD–PixE complex has a molecular mass of ~400 kDa (Yuan and Bauer 2008). Given that the molecular masses of monomeric PixD and PixE are 18 and 43 kDa respectively, a complex of ~400 kDa would most likely be composed of ten PixD and five PixE molecules. This ratio of PixD to PixE molecules seems reasonable, because each PixE molecule could interact with an interface formed by an α-β-γ-χ set of nearest-neighbor subunits on the outside of the decamer. However, Ren and co-workers, having used blue-native PAGE to determine the molecular mass and oligomeric state of the PixD–PixE complex, concluded that the PixD–PixE complex contained a PixD decamer and up to four PixE molecules (Ren et al. 2013). They first examined the equilibrium state of PixD and found two bands on the gel with molecular masses of ~40 and ~180 kDa, suggesting an equilibration between a dimer and a decamer in agreement with the size-exclusion chromatography study of PixD described above. When they added purified PixE into a solution of purified PixD, four bands were present in the gel with molecular masses of 220, 260, 300, and 340 kDa; these molecular masses correspond to calculated masses for PixD_10_–PixE_1_ (215 kDa), PixD_10_–PixE_2_ (258 kDa), PixD_10_-PixE_3_ (301 kDa), and PixD_10_–PixE_4_ (344 kDa). When the amount of PixE added into the PixD solution was increased, the equilibrium shifted so that eventually only the 344-kDa band was present. In addition, a purified PixD–PixE complex, co-expressed in *E. coli*, ran as a 344-kDa band on blue-native PAGE (Ren et al. 2013). From these observations, the authors proposed that the PixD–PixE complex is composed of a PixD decamer and four PixE monomers. The authors also performed docking simulations of the PixD decamer and a PixE monomer, and the results suggested that a PixE monomer docks between two PixD monomers on the top of a pentameric ring (Fig. 5) (Ren et al. 2013). This docking study suggested that there are four possible PixE-binding sites**,** with two on each tetrameric ring of the PixD decamer.

Yuan and co-workers determined a crystal structure for a PixD mutant in which Tyr8 had been replaced by a phenylalanine (Yuan et al. 2011). The structure of this mutant was an asymmetric oligomer composed of six subunits arranged such that two PixD trimers were stacked face to face. Biochemical and genetic studies have shown that this mutant is locked in a light-signaling state conformation even when not excited by blue light (Masuda et al. 2008). Possibly this hexameric quaternary structure represents a state mimicking the transition state between the light-signaling and dark states. The geometry of the PixD_10_–PixE_4_ complex shown in Fig. 5 supports this hypothesis, because formation of stacked trimeric PixD semicircles during dissociation of the pentameric rings would remove a PixE monomer from a PixD pentamer. Additional structural studies, e.g., cryo-electron microscopy of a PixD–PixE complex, would be necessary to determine the exact structure of the complex.

What is the physiological meaning of complex formation between PixD and PixE? Tanaka and co-workers applied the transient-grating technique to monitor interaction dynamics between PixD and PixE. Disassembly of PixD_10_–PixE_5_ (or PixD_10_–PixE_4_) took place only after simultaneous photoexcitation of two PixD subunits in a complex; excitation of only one PixD subunit was insufficient to dissociate a PixE monomer(s) from the PixD–PixE complex (Tanaka et al. 2012). These observations suggested that oligomerization of PixD causes its functional response to be light-intensity dependent, although the correlation between the response and light intensity was not linear. Perhaps, as a consequence of evolutionary pressures, the equilibrium state between the PixD dimer and decamer has been optimized to function as a light-intensity sensor to control *Synechocystis* phototaxis.

### Regulation of phototaxis by PixD–PixE complex formation

*Synechocystis* mutants lacking PixD (ΔPixD), PixE (ΔPixE), or both proteins (ΔPixDE) have been genetically characterized to determine the significance of the light-dependent assembly/disassembly of the PixD–PixE complex (Sugimoto et al. 2017). The ΔPixD mutant showed negative phototaxis even though the wild-type strain showed positive phototaxis (Masuda and Ono 2004; Okajima et al. 2005). Conversely, mutants ΔPixE and ΔPixDE showed positive phototaxis in the same manner as the wild-type strain (Sugimoto et al. 2017). These results clearly indicate that PixE functions downstream of PixD and prevents positive phototaxis. Therefore, formation of PixD_10_–PixE_5_ or PixD_10_–PixE_4_ inhibits PixE function. As noted above, upon photoexcitation of two PixD molecules in a complex, PixE was released from the complex and positive phototaxis was inhibited. Wild-type *Synechocystis* exhibits negative phototaxis only under intense blue-light irradiation (100 μmol m^−2^ s^−1^; Sugimoto et al. 2017), which supports the hypothesis that PixD is a light-intensity sensor rather than simply a light sensor (Tanaka et al. 2012).

Recently, high-resolution imaging of *Synechocystis* phototaxis was performed (Schuergers et al. 2016), which revealed that the bacterium acted as a spherical microlens by focusing directional light at its cellular edge that is distal to the light source. Furthermore, involvement of type-IV pili for PixD/PixE-dependent negative phototaxis has been characterized (Nakane and Nishizaka 2017). Upon lateral irradiation with blue light, cells undergo a typical type-IV pili extension cycle, i.e., pili attachment to a surface, followed by retraction; this cycle allows the cells to move away from the blue-light source via a twitching motility. Motor proteins, including PilB, control the extension and retraction of type-IV pili and are located in a region of the cell such that they can control the direction of motion (Schuergers et al. 2015). In other words, the location of PilB determines the direction of cell movement. These results suggest that the PixD–PixE complex regulates PilB localization, although how the complex does this is not known. Characterization of the location(s) of PixD and PixE during phototaxis should help delineate the PixD-dependent light-signal transduction mechanism(s) that controls the type-IV pili-dependent phototaxis response.

## Conclusions and perspectives

Since the discovery of BLUF proteins 15 years ago, the photoreceptors have been used as a model to understand how exposure to light can modulate protein structure. By studying BLUF proteins, a unique mechanism of light-signal propagation has been uncovered, which involves a proton-coupled electron transfer that induces rearrangement of the hydrogen-bond network in the flavin-binding pocket. This small structural alteration results in dynamic changes between FAD and the apoprotein, which is propagated as large conformational changes between the BLUF domain and its downstream factor(s). Proteins functioning downstream of PixD, which controls the phototaxis response of the cyanobacterium *Synechocystis* sp. PCC6803, have been identified. Further characterization of the BLUF photoreceptors and proteins with which they interact should provide crucial information concerning how their inter- and intramolecular structures are rearranged by the light signal to cause a related physiological response. Such information will be useful for the rational design of new optogenetic tools for synthetic biology and other applications (Masuda 2013).
